# Near-infrared laser delivery of nanoparticles to developing embryos: A study of efficacy and viability

**DOI:** 10.1002/biot.201000205

**Published:** 2011-05

**Authors:** Jose Umanzor-Alvarez, Emily C Wade, Aliya Gifford, Kankowan Nontapot, Ariana Cruz-Reese, Tetsuya Gotoh, Jill C Sible, Giti A Khodaparast

**Affiliations:** 1 Department of Physics, Virginia Polytechnic Institute and State UniversityBlacksburg, VA, USA; 2 Department of Physics, Norfolk State UniversityNorfolk, VA, USA; 3 Department of Biological Sciences, Virginia Polytechnic Institute and State UniversityBlacksburg, VA, USA

**Keywords:** Development, Methods, Quantum dots, Two photon optical process, *Xenopus laevis*

## Abstract

Targeted delivery of materials to individual cells remains a challenge in nanoscience and nanomedicine. Near infrared (NIR) laser injection may be a promising alternative to manual injection (where the micropipet diameter limits targeting to small cells) or other laser techniques (such as picosecond green and UV lasers, which can be damaging to cells). However, the efficiency with which NIR pulses can deliver nanoparticles and any adverse effects on living cells needs thorough testing. Toward this end, we have determined the efficacy and toxicity of delivering quantum dots (QDs) into cells of *Xenopus laevis* embryos by NIR laser injection. Because this model system provides not only living cells but also a developing organism, we were able to assess relatively long-term effects of NIR pulses on embryonic development (through the tadpole stage). We developed parameters for NIR pulses that did not affect embryonic viability or morphology and delivered QDs as effectively as manual injection. Higher intensities of NIR pulses caused permanent damage to the targeted cells, and thus NIR pulses may also prove useful for ablation of specific cells within tissues.

## 1 Introduction

Advances in molecular biology and imaging provide unprecedented opportunity to investigate physiological events and treat diseases at the level of the individual cell. However, one continuing challenge is the targeting of DNA, nanoparticles and other molecules to specific cells without damaging these cells. On the other hand, a technique that could destroy individual cells within a tissue would provide a powerful tool to study cell fate and function and to eliminate pathological cells without damaging their healthy neighbors.

Manual microinjection of QDs into large cells using microsyringes has already been demonstrated but this technique is impractical for small physical dimensions such as bacterial cells. The development of a reliable and reproducible means to inject nanoprobes into living cells will extend our knowledge of cell function and advance biomedical and environmental research. QDs as nanoprobes exhibit unique optical properties due to their electronic states where the QD fluorescence can be confined to the progeny of the injected cell [1].

Near infrared (NIR) microinjection technique is a promising alternative to the existing methods of delivering materials to living cells such as injection by pipettes, biolistics, and electroporation [2, 3]. Recently, Tirlapur and König demonstrated a micro-injection technique that employed tightly focused NIR pulses to create pores and inject DNA in cell membranes with very high cell survival rates [3–5]. In this method, several cells can be targeted rapidly, which should be valuable for screening the effect of drugs and biomolecules on living cells.

König et al. also proposed a nanosurgery tool using NIR pulses to perform dissection of chromosomes [6]. Maxwell et al. [7] also have demonstrated dissection of individual mitochondria in a living cell without damaging the rest of the cell on a scale of a few hundred nanometers using the femtosecond tightly focused NIR laser pulses at a low repetition rate (1 kHz) [7, 8]. Focused NIR femtosecond pulses (using high numerical aperture objectives) can be confined to a small focal volume resulting in high photon densities to induce multiphoton absorption at the laser focus, even in materials that are normally transparent. Using NIR pulses with duration of ∼100 fs, only a few nanojoules of laser energy are necessary for manipulation and dissection.

The advantages of using NIR femtosecond pulses include: low absorption of melanin and hemoglobin in the NIR region such that the laser light can penetrate into tissues, capability of imaging and probing biological structures and processes with a submicrometer spatial resolution. In addition, this method provides the possibility of nanosurgery of individual organelles and disruption of living cells, where low energy laser pulses cause only negligible damage around the laser focus [2–5].

Use of nanosecond laser pulses from a frequency-tripled Nd/YAG source has also been tried, but this technique permanently damages the cells. Use of focused continuous wave or long-pulse lasers in visible and ultraviolet has several disadvantages such as small penetration depth, strong absorption, and damage outside the focal volume. Dissection using picosecond green laser systems and UV lasers require energies that are two to three orders of magnitude higher than NIR pulses [9]. NIR photons demonstrated a longer penetration depth, higher resolution images, and reduced photo- and thermal damage in which water is the major NIR absorber.

Better understanding of the NIR nanoprocess-ing of cellular structures without disturbing adjacent layer is important to the development of new techniques in cell biology. Recently, the interaction of femtosecond NIR lasers in single living BAEC cells to create pores has been examined [10].

**Figure 1:**
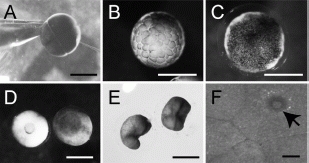
Low-power images of *X. laevis* embryos. (A) Four-cell embryo, 2 h pf. Embryo is being microinjected into one cell, a dorsal blastomere. (B) 32-cell embryo, 4 h pf. (C) Late blastula stage embryo, 9 h pf. (D) Gastrulating embryos, vegetal view (left), animal view (right), ∼14–15 h pf; (E) tailbud stage embryo prior to hatching, ∼24 h pf; (F) high-power view of embryo immediately after laser targeting for 1 s. Arrow indicates targeted region. In an experiment with 10 embryos, 80% of embryos targeted with similar settings as the measurement presented in Fig. 1 F survived through the swimming tadpole stage with no observable abnormalities. Scale bars = 1 mm (A–E), 100 μm (F).

Here, we report the interaction of *X. laevis* with NIR radiations as well as the injection of quantum dots (QD) using a two-photon confocal microscope. In order to determine the efficacy and potential toxicity of delivery of nanoparticles to living cells *via* NIR pulses, we performed studies using *X. laevis* embryos. Eggs and embryos of *X. laevis*, the South African clawed frog, are a classic model system for investigating questions about cell division and cell fate. Because *X. laevis* eggs are large (∼1.5 mm in diameter), they can be manipulated conveniently by manual micro injection of RNAs, drugs, and other molecules (Fig. 1A; for a review, see Sible and Wroble [11]). Upon fertilization, *X. laevis* eggs divide by a series of cleavage events (Fig. 1) and reach the midblastula stage with approximately 4000 cells at ∼6 h post-fertilization (pf).The embryos then enter the midblastula transition where cells become motile and embryonic transcription initiates. They gastrulate (where the initial stages of cell differentiation occur) at approximately 10 h pf (Fig. 1).

Meticulous lineage analysis studies in which tracers have been injected into individual cells have enabled scientists to produce developmental fate maps for individual cells in the intact embryo [12], and more recent studies in *X. laevis* indicate that QDs can be a valuable tool for further refined studies of morphogenesis [1, 13]. The combination of size, rapid development, and mapping of cell fates renders the *X. laevis* embryo an excellent model system in which to test new technologies for delivering and detecting nanoparticles.

In this study, we probed the effect of NIR pulses on the development of *X. laevis* embryos through the tadpole state. We developed conditions suitable for delivery of QD nanoparticles to living cells *via* NIR pulses and compared these to manually injected embryos. Our results suggest that NIR pulses hold promise for targeted delivery of nanoparticles, and at higher intensities, NIR pulses can destroy targeted cells in a living tissue.

## 2 Materials and methods

### 2.1 Fertilization, manual microinjection, and brightfield visualization of embryos

All studies with *X. laevis* were approved by the Institutional Animal Care and Use Committee at Virginia Tech. Eggs from wild-type *X. laevis* (South African clawed frog; Xenopus Express) were fertilized in vitro, dejellied in 2% cysteine, 0.1X MMR (0.5 mM HEPES, pH 7.8, 10 mM NaCl, 0.2 mM KCl, 0.1 mM MgSO_4_,0.2 mM CaCl_2_,0.01 mM EDTA,), and maintained in 0.1X MMR. Embryos were staged according to Nieuwkoop and Faber [14]. Embryos at the indicated stage were placed in glass dishes containing 5% Ficoll in 0.1X MMR, and individual cells were injected with ∼5 nl QDs (520 nm Adirondack Green CdSe/ZnS T2-MP Non-functionalized EviTags) using a Harvard Apparatus PLI-100 microinjector and glass micropipettes pulled with a Narishige PC 10 pipette puller. Embryos were transferred to and maintained in 0.1X MMR with 5% Ficoll for approximately 2 h after injection. Embryos were manipulated and visualized at low power using an Olympus SZX12 stereomicroscope and photographed using an Olympus DP 10 digital camera. For some experiments, embryos were placed at 16°C between observations, which slows development. For simplicity, the times pf reported here were temperature-compensated to reflect the time embryos would have reached the relevant developmental stage at room temperature (21°C).

### 2.2 NIR viability studies

Embryos at the two-cell stage were targeted with NIR laser pulses with a 80 MHz repetition rate from a Titanium-Sapphire (Ti/S) laser emitting 100 fs pulses between 750 and 850 nm (with maximum average output power of ∼900 mW). The laser beam was focused on cells to about 100 μm diameter, with an average power of ∼25 mW (pulse energy of ∼0.25 nJ). After targeting, embryos were maintained in 0.1X MMR, observed periodically for viability, and representative embryos with normal or abnormal morphology were photographed.

### 2.3 NIR delivery of nanoparticles via two-photon confocal microscopy

The considerable advantages of two-photon excitation microscopy arise from the basic principle that the absorption depends on the square of the excitation intensity. Two-photon excitation is generated by focusing laser pulses through the microscope optics. As the laser beam is focused, the photon density and the probability of two excitation increases. The laser focal point is the only location along the optical path with a large photon density to generate significant occurrence of two-photon excitation. A pinhole conjugated to the focal plane obstructs the light coming from objects outside that plane; therefore, only light reflected or emitted from in-focus objects can reach the detector.

Here, individual embryos at the indicated stages were placed in glass bottom chambers (MatTek P35G-1.5-20-C) containing a drop composed of 3 μl QDs (520 nm Adirondack Green CdSe/ZnS T2-MP non-functionalized EviTags) mixed with 20 μl 0.1X MMR. The liquid covered approximately the bottom half (vegetal side) of the embryo. A Zeiss laser scanning microscope (LSM), model 510, with a 10X objective and a 660 nm reflector (only reflects wavelengths >660 nm), was tuned to 700 nm where only 10–12% intensity of 30 nJ laser pulses were used to scan the embryo and position the laser on a region of interest (ROI) with a diameter of 100–200 μm. QDs were then delivered to ROI by scanning the ROI at 700 nm, 100% intensity for 1 s. After injection, embryos were rinsed several times in 0.1XMMR solution to remove any QDs attached to the outside of the embryo.

### 2.4 Visualization of QDs in embryos

Embryos injected with QDs were visualized at low power using an Olympus SZX12 stereomicroscope on a SZX-ILLD100 illumination base with an SZX-RFL2 coaxial filter attachment and an SZX-MGFP filter and an Olympus Color View 12 digital camera. Confocal microscopy was performed with a Nikon or Zeiss confocal microscope as indicated. In order to take images using the Nikon microscope, an argon laser with UV (UV-2A EX 330–380 DM 400 BA 420), blue (FITIC EX 450-460 DM 505 BA 520) and green (G-2E/C TRITIC EX 540/570 DM 575 BA 605) filters were used. In the Zeiss microscope, laser emissions from an Argon laser, fixed at 514 nm and fluorescence filters cubes (FITC/GFP: 450–490 nm excitation, 515–586 nm emission), were used to take the images.

## 3 Results and discussion

### 3.1 *X. laevis* embryos can be targeted by NIR with minimal damage

We probed the impact of NIR lasers on the viability and development of *X. laevis* embryos. We studied the interaction of the laser pulses with the frog embryos as a function of wavelength, power, and exposure time. We identified an optimum configuration of 100 μm diameter and an average power of 25 mW (pulse energy of 0.25 nJ) that allowed us to create pores in the cells without damaging them. Figure 1F shows the high-power view of an embryo immediately after laser targeting for 1 s to deliver a solution of QDs.

**Figure 2:**
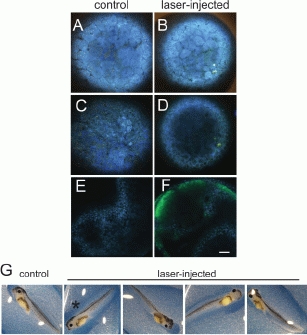
Delivery of QDs at 1 s exposure and subsequent embryonic development. Control embryos (A,C,E) or embryos targeted with QD solution (B,D,F) for 1 s at 700 nm with an ROI of 100 μm were photographed at 3 h (A,B), 6 h (C,D), and 24 h (E,F). QDs fluoresce green and autofluorescence of embryos is blue. (G) A control embryo and four targeted embryos were photographed at 72 h using a stereoscope the tadpoles are alive and swimming, where the views are from different angles. The embryo indicated by an asterisk (*) is the same embryo shown in (B,D,F). Scale bar = 100 μm in A–F. A fifth targeted embryo died after 24 h. On three different experimental trials involving 40 embryos, a survival rate of more than 60% was observed over a time span of 50 h.

Although the site of delivery is detectable immediately after targeting, embryos treated with these settings recover. Figure 2B, D, and F follows the embryos shown in Fig. 1F through the tailbud stage. Development is normal when compared to a sibling control (Fig. 2A, C, and E). Typically 80% of embryos targeted using these conditions survive through the swimming tadpole stage with no observable abnormalities.

Based on multiple trials under different conditions, we conclude that embryos can safely be targeted with the selected settings for 1 s. Higher doses with a similar laser intensity may be damaging and perhaps suitable to cell ablation applications.

### 3.2 Nanoparticle quantum dots can be delivered by NIR

**Figure 3:**
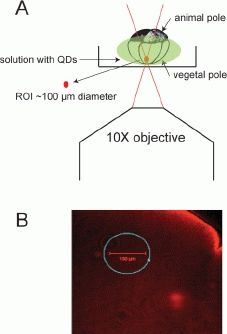
Delivery of QDs *X. laevis* embryos *via* NIR. (A) Schematic of laser targeting approach. Embryos in a dish containing a solution of QDs were first scanned at low power (700 nm; 10–12% intensity) to target a single cell in the vegetal pole since this side faces down and the incident laser comes up through the bottom of the dish. A ROI of 100 μm was then targeted for 1 s at 700 nm. (B) Low-power scan of an embryo showing the ROI. A 660 nm reflector, which only reflects wavelengths >660 nm, were used.

In the second set of studies, we attempted to deliver colloidal QDs into the *X. laevis* embryos by NIR pulses. In this study, use of the two-photon confocal microscope was pivotal. Two-photon excitation allows for the use of longer, less damaging, wavelengths of light, while still achieving excitation energies that would emit at half of that wavelength. This property allows us to use NIR pulses (which are relatively harmless to living cells) in place of UV light (which is damaging to live cells). NIR laser pulses from a Ti/S oscillator, attached to the Zeiss microscope, were tuned at 700 nm and passed through a long pass filter before entering the microscope. QDs diffuse into a cell of the frog embryo *via* the pores made by the laser pulses. In a confocal microscope, a pinhole conjugated to the focal plane obstructs the light coming from objects outside that plane; therefore, only light reflected or emitted from in-focus objects can reach the detector. Focused NIR femtosecond pulses (using high numerical aperture objectives, in our case 10X) can be confined to a small focal volume resulting in high photon densities to induce multiphoton process resulting in absorption even in materials with are transparent in NIR. The specific set up we used is diagrammed in Fig. 3A and detailed in the Materials and Methods section. Figure 3B shows an image captured and the ROI targeting feature of the Zeiss LSM.

**Figure 4:**
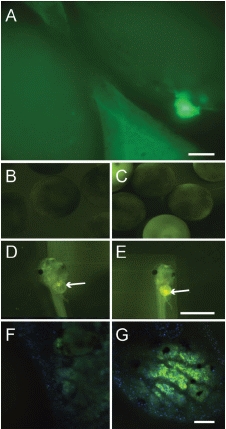
Low and high power views of embryos after laser injection with QDs. (A) An uninjected (left) and laser-injected (right) embryo right after injection. (B) Control- and (C) laser-injected embryos photographed at the early gastrula stage, ∼8 h after injection. (D) Control- and (E) laser-injected embryos photographed at the tadpole stage, ∼3 d after injection. Note some nonspecific autofluorescence in the gut (arrows) of the control embryo but a higher level of fluorescence in the injected embryo. (F) Control- and (G) laser-injected embryos ∼2 days pf photographed in the gut region using the Nikon confocal microscope. Scale bars = 100 μm in A, F, and G and ∼1 mm in B –E. Under similar experimental conditions, four laser and one manually injected embryos with QDs were monitored and photographed, suggesting a higher level of fluorescence in the guts.

The uptake of QDs in embryos was verified by both low and high power fluorescence microscopy (Fig. 4). Immediately after laser-targeting, QDs could be observed in targeted embryos in a region ∼100 μm in diameter, the size of the selected ROI (Fig. 4A). As embryos developed, fluorescence could be observed in some of the targeted embryos (depending on their orientation in the photograph) via low power fluorescence microscopy relative to uninjected or water-injected control embryos (Fig. 4B and C). However, it is important to note that the images shown were some of the better examples. *X. laevis* embryos are known for their high degree of autofluorescence over a range of wavelengths [15], and frequently the background fluorescence in the control embryos made it difficult to appreciate the QDs in the targeted embryos. By the tadpole stage, much of the fluorescence appeared to be targeted to the gut. Even though the gut exhibited some autofluorescence at low power (Fig. 4D), enhanced fluorescence above this background was detected in laser-targeted embryos (Fig. 4E). When viewed by confocal microscopy, the accumulation of QDs in the gut of targeted embryos was more apparent compared to control embryos (Fig. 4F and G).

**Figure 5:**
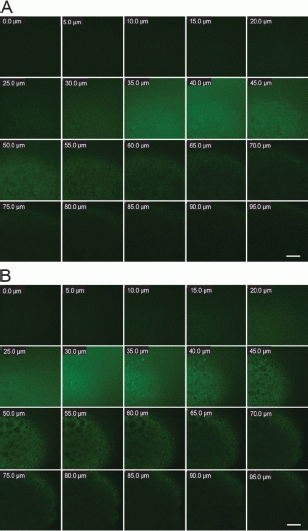
Z-stack series to visualize QDs in laser-targeted embryos. QDs were delivered to embryos at the 32–64-cell stage, and embryos were imaged using the Zeiss LSM at the 32–64 cell stage. Uninjected (A) and laser-targeted (B) embryos were imaged at 5 μm intervals through a 100 μm section in the center of the embryo. The Z-Stack images presented here demonstrate an example of six embryos injected by QDs, using the NIR pulses, under similar experimental conditions.

To better appreciate the localization of the QDs in laser-target embryos *versus* the background autofluorescence inherent in *X. laevis* embryos, a Z-stack series was generated with uninjected and laser-targeted embryos (Fig. 5). QDs were delivered to embryos at the 32–64 cell stage, and embryos were imaged using the Zeiss LSM at the 32–64 cell stage. Although both series possess frames with autofluorescence, the QD-targeted embryos have a more definite pattern of punctate, intensely staining material, and suggestion of subcellular localization (nuclear exclusion) of the QDs.

## 4 Conclusions

Taken together, these data indicate that QDs delivered by NIR can be taken up by and persist in *X. laevis* embryos without damaging them. The methodology appears comparable to the well-established technique of manual microinjection with the advantage that the ROI can be precisely controlled and should be adjustable for delivery of nanoparticles to much smaller cell types. Multiple cells can rapidly be targeted by either steering the beam or translating the samples. NIR manipulation also offers the promise of targeted cell ablation by adjusting the exposure time and intensity to a level that kills the cells. The latter application will be of value both in nanomedicine and in more refined studies of cell fate.

## Abbreviations

LSM: laser scanning microscope
NIR: near infrared
pf: post-fertilization
QDs: quantum dots
ROI: region of interest
